# Remote Ischemic Conditioning to Protect against Ischemia-Reperfusion Injury: A Systematic Review and Meta-Analysis

**DOI:** 10.1371/journal.pone.0042179

**Published:** 2012-07-31

**Authors:** Daniel Brevoord, Peter Kranke, Marijn Kuijpers, Nina Weber, Markus Hollmann, Benedikt Preckel

**Affiliations:** 1 Laboratory of Experimental Intensive Care and Anesthesiology, Academic Medical Center, Amsterdam, The Netherlands; 2 Department of Anesthesiology, Academic Medical Center, Amsterdam, The Netherlands; 3 Department of Anesthesia and Critical Care, University Hospital Würzburg, Würzburg, Germany; S.G. Battista Hospital, Italy

## Abstract

**Background:**

Remote ischemic conditioning is gaining interest as potential method to induce resistance against ischemia reperfusion injury in a variety of clinical settings. We performed a systematic review and meta-analysis to investigate whether remote ischemic conditioning reduces mortality, major adverse cardiovascular events, length of stay in hospital and in the intensive care unit and biomarker release in patients who suffer from or are at risk for ischemia reperfusion injury.

**Methods and Results:**

Medline, EMBASE and Cochrane databases were searched for randomized clinical trials comparing remote ischemic conditioning, regardless of timing, with no conditioning. Two investigators independently selected suitable trials, assessed trial quality and extracted data. 23 studies in patients undergoing cardiac surgery (15 studies), percutaneous coronary intervention (four studies) and vascular surgery (four studies), comprising in total 1878 patients, were included in this review. Compared to no conditioning, remote ischemic conditioning did not reduce mortality (odds ratio 1.22 [95% confidence interval 0.48, 3.07]) or major adverse cardiovascular events (0.65 [0.38, 1.14]). However, the incidence of myocardial infarction was reduced with remote ischemic conditioning (0.50 [0.31, 0.82]), as was peak troponin release (standardized mean difference −0.28 [−0.47, −0.09]).

**Conclusion:**

There is no evidence that remote ischemic conditioning reduces mortality associated with ischemic events; nor does it reduce major adverse cardiovascular events. However, remote ischemic conditioning did reduce the incidence of peri-procedural myocardial infarctions, as well as the release of troponin.

## Introduction

Organ ischemia causes tissue damage in a variety of clinical settings, such as acute infarctions and/or hypoperfusion during surgery and organ transplantations. Restoration of adequate blood flow is the main treatment. However, even after restoration of blood flow tissue damage continues, partly induced by reperfusion itself. Research into the reduction of ischemia-reperfusion injury has gained an impulse after the discovery of ischemic preconditioning in 1986 by Murry et al. [1] In ischemic preconditioning, a series of short non-lethal periods of ischemia interspersed with reperfusion periods, renders a target organ more resistant against a subsequent ischemic event. In addition, ischemic postconditioning, e.g. interrupted reperfusion or low-flow reperfusion, results in a reduction of damage compared to full-flow reperfusion. [2]

Remote ischemic conditioning is an approach of conditioning in which not the target organ, such as the heart, but instead a more accessible tissue is submitted to a conditioning stimulus. First discovered to offer protection within one organ, [3] remote preconditioning has been found to offer protection against ischemia reperfusion injury also when a different organ, or even skeletal muscle tissue is used for conditioning. [4] Postconditioning is also possible with remote conditioning, offering the possibility of protecting patients suffering from unpredictable ischemic events, such as acute infarctions. [5]

There is an increasing number of clinical studies investigating possible protection by remote ischemic conditioning, mostly in cardiac and major vascular surgery. These studies show a reduction of the release of biomarkers like serum troponine values. The release of troponine is related not only to myocardial infarction, but also to other cardiovascular events in the post-operative period.

In 2008 Takagi et al. conducted a first meta-analysis of the four available studies, with a total of 184 patients undergoing cardiovascular surgery. [6] They found that remote ischemic preconditioning reduced the release of biomarkers of myocardial injury, however, no analysis on adverse events was done. We performed a systematic review of an increased number of randomized trials investigating remote ischemic conditioning to answer the question whether remote ischemic conditioning, either before or during ischemia, improves the outcome of patients suffering from acute ischemia or who are at risk of developing ischemia during surgery.

## Methods

### Search Strategy and Selection of Papers

Medline, EMBASE and Cochrane databases were searched independently by two investigators (DB, MK) using the following terms: “remote preconditioning”, “remote postconditioning”, “remote perconditioning”, “remote conditioning”, “remote ischemic preconditioning”, “remote ischemic postconditioning”, “remote ischemic perconditioning”, “remote ischemic conditioning”, “remote ischaemic preconditioning”, “remote ischaemic postconditioning”, “remote ischaemic perconditioning” and “remote ischaemic conditioning.” Literature was searched until July 2011, no search limitations were used and all found abstracts were screened for eligibility. Abstracts of papers identified through other sources were screened as well. Eligible trials were those in which patients suffering from, or being at risk for, ischemia were randomly assigned to receive either remote ischemic conditioning, regardless of the timing or the way the stimulus was induced, or no ischemic conditioning. Full paper manuscripts were obtained of all selected articles based on the assessment of abstracts. Only fully published trials were included, abstracts and congress presentations were not included. Primary outcome to be assessed was mortality; secondary outcome parameter was the combined endpoint of major adverse cardiovascular events, which was defined as stroke, myocardial infarction, atrial fibrillation, or kidney injury. Additional secondary endpoints were length of stay in the hospital and in the intensive care unit, biomarkers of cardiac and kidney injury, and kidney function. Trials not reporting any of these parameters were excluded from the review. When the independent search led to papers included by only one author, this was discussed to reach consensus. Two authors independently reviewed all full reports that could possibly meet inclusion criteria, guided by the Cochrane Handbook for Systematic Reviews and the PRISMA statement. [7], [8] In case of disagreement, consensus was sought by discussion with a third author.

### Reviewing and Data Extraction

All selected papers were reviewed by two investigators (DB, MK), who were not blinded for the origin of the paper, nor the authors. Both investigators independently extracted data to a data extraction sheet. Data on the study population, in- and exclusion criteria, control and intervention protocol, randomization, blinding and follow-up were collected, as well as the outcome parameters mentioned above. Outcome parameters that were reported in the paper but were not the focus of this review were not extracted. Concerning the timing of events such as mortality, we used peri-operative or in-hospital event rate, if reported. For clinical parameters such as the occurrence of myocardial infarction or kidney injury, the criteria of the respective paper were used. We did not extract data on myocardial infarction from studies that were done in patients suffering from myocardial infarction.

With respect to biomarker data, we used the peak values as reported in the paper. We renamed both troponin-I and troponin-T “troponin”, and to compensate for the different types of troponin as well as the variation induced by different methods of analysis we used the standardized mean difference for analysis. If the data was presented in a graph, but not in text, we requested the corresponding author of the paper to provide these data. If these data were not provided we extrapolated it from the graph if the scale allowed a sufficiently precise estimation, i.e. if the scale and resolution of the graph allows the extraction of the graph without ambiguity. Inconsistency of the extracted data was discussed among the investigators, clarified and accounted for.

After extraction of relevant data to the data sheets by the two investigators, these were checked for inconsistencies, which were then resolved by discussion and joint reviewing of the paper. In case consensus couldn’t be reached, a third investigator would be consulted (BP).

Papers were assessed for risk of bias by DB and MK on the following areas: randomization sequence generation, concealment of randomization sequence, blinding of intervention, blinding of outcome assessment, incomplete outcome reporting, and were classified as having low risk, high risk or unclear risk of bias for each item, as suggested in the Cochrane Handbook. [7]

### Data Analysis

The verified data were analyzed using Review Manager (Version 5.1). [9] DB entered the data, MK verified data entry. The odds ratio (Mantel-Haenszel), mean difference (inverse variance) and their corresponding 95% confidence intervals (95% CI) were calculated for dichotomous or continuous outcome data, respectively. A fixed-effect model was used in case of no relevant statistical heterogeneity. Statistical heterogeneity was assessed with the I^2^-test and assumed when I^2^ was >25%. A random-effect model was used in all other cases. A significant effect of an intervention was assumed if the 95% CI did not include the value 1.0 for odds ratio or 0 for mean difference. When the standard error was reported but not the standard deviation, the standard deviation was calculated. If continuous data was reported as median and range, we calculated an estimation of the mean and standard deviation as described by Hozo et al. [10] When not the range, but the interquartile range was reported, we assumed this to be 1.35 standard deviations.

Studies containing zero events in both arms are excluded when calculating an odds-ratio. To avoid over-estimating a possible effect due to the exclusion of patients in studies with no events, we recalculated the odds-ratio of dichotomous outcomes after adding one event in each arm, allowing us to include the patients in those studies.

Forrest plots were used for graphical presentation, as well as an l’Abbé plot for dichotomous endpoints. We intended to perform a sub-group analysis for different patient populations if more than four studies were available: cardiac surgery, coronary artery bypass grafting surgery, valve-surgery, pediatric cardiac surgery and percutaneous coronary intervention (acute and elective).

## Results

### Description of Studies

#### Search results

**Figure 1:**
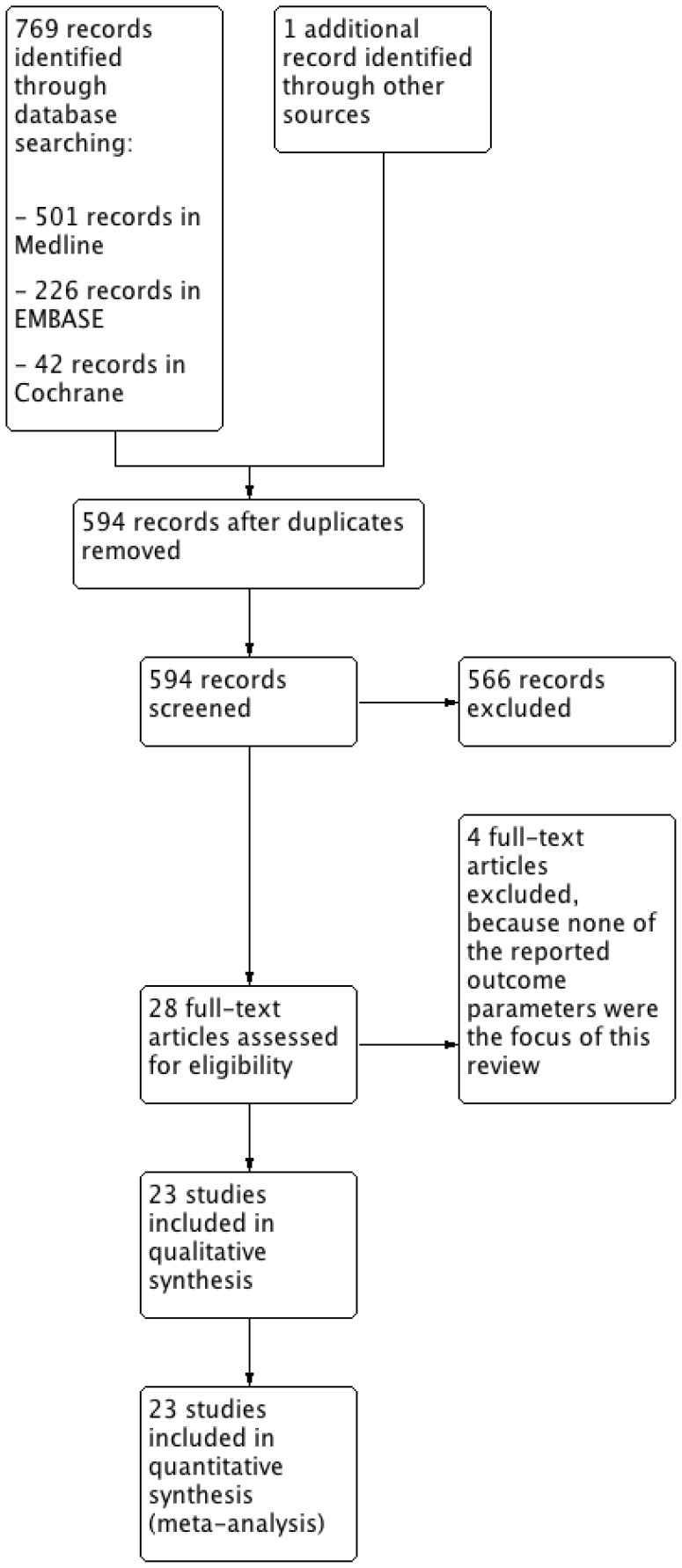
Flow-schedule of search and selection of studies.

Searching resulted in 501 hits in Medline using PubMed, 226 hits in EMBASE and 42 hits in the Cochrane data base of clinical trials (see Figure 1), as well as one additional abstract identified through other sources. After checking for duplicates, 594 unique references remained. All abstracts were screened and 28 papers were identified for further evaluation, which were obtained in full print. Reading the full prints resulted in the direct exclusion of two papers, [11], [12] and after discussion between the three investigators of another two, [13], [14] that did not report any of the outcome parameters, which left 24 papers to be included for review and analysis (Table 1). One paper [15] provided additional data on kidney injury in patients of two previously published studies, [16], [17] thus, 23 clinical studies were finally analyzed.

**Table 1:** Overview of included studies.

Study	Patientpopulation	Number of pat.	Groups	Form of remote conditioning	Endpoints	Results
Ali 2007 [34]	Open AAA* repair	82	Remote ischemic preconditioning VS control	Leg ischemia by clamping of the common iliac artery, 10 minutes left and 10 minutes right	Myocardial injury, asdefined by an increase inserum cardiac troponin>0,40 ng/ml Sec:myocardial infarction,kidney injury, death	Preconditioning reduced the risk of myocardial injury
Ali 2010 [21]	On-pump CABG^†^	100	Remote ischemic preconditioning VS control	Arm ischemia and reperfusion, by inflation of a cuff at 200 mmHg placed at the arm, for 3×5 minutes	CK-MB release	Remote ischemic conditioning reduced release of CK-MB postoperatively
Bøtker 2010 [33]	Primary PCI^§^	333	Remote ischemic perconditioning VS control	Arm ischemia and reperfusion, by inflation of a cuff at 200 mmHg placed at the arm, for 4×5 minutes	Salvage index at 30 days measured by scintigraphy Sec.: Infarct size, troponin levels, death, reinfarction, hospitalization for heart-failure, LVEF^§^	Remote ischemic conditioning increased salvage index, when corrected for ventricle size
Cheung 2006 [25]	Heart surgery for congenital defects	37	Remote ischemic preconditioning VS control	Leg ischemia and reperfusion, by inflation of a cuff at systolic pressure +15 mmHg placed at the leg, for 4×5 minutes	Troponin levels, mixedvenous saturation, urineoutput, inotropicdemand, lung function,systemic inflammatory response	Remote ischemic conditioning reduced myocardial injury
Choi 2011 [29]	Complex valve surgery	76	Remote ischemic preconditioning VS control	Leg ischemia and reperfusion, by inflation of a cuff at 250 mmHg placed at the leg, for 3×10 minutes	Kidney injury, asdefined by in increase inserum creatinine bymore than 50% or0.3 mg/dl(26.5 umol/L). Sec:CK, CK-MB levels	Remote ischemic preconditioning did not reduce kidney or myocardial injury
Günyadin 2000 [18]	On-pump CABG	8	Remote ischemic preconditioning VS control	Arm ischemia and reperfusion, by inflation of a cuff at 300 mmHg placed at the upper arm, for 2 cylci of 3 minutes ischemia and 2 minutes reperfusion	CK-MB and LDH release	Remote ischemic conditioning increased biomarker release
Hausenloy 2007 [16]	On-pump CABG	66	Remote ischemic preconditioning VS control	Arm ischemia and reperfusion, by inflation of a cuff at 200 mmHg placed at the arm, for 3×5 minutes	Troponin release	Remote ischemic conditioning reduced troponin release
Hong 2010 [38]	Off-pump CABG	133	Remote ischemic preconditioning VS control	Arm ischemia and reperfusion, by inflation of a cuff at 200 mmHg placed at the arm, for 4×5 minutes	Troponin release	Remote ischemic preconditioning did not significantly reduce troponin release
Hoole 2009 [31]	Elective PCI	242	Remote ischemic preconditioning VS control	Arm ischemia and reperfusion, by inflation of a cuff at 200 mmHg placed at the arm, for 3×5 minutes	Primary: Troponinlevels at 24 hoursSecondary: serumcreatinine, CRP,estimated GFR. MACE^||^rate at 6 months	Remote ischemic preconditioning reduced troponin levels
Iliodromitis 2006 [30]	Elective PCI	41	Remote ischemic preconditioning VS control	Arm ischemia and reperfusion, by inflation of a cuff at 200 mmHg placed at both arms, for 3×5 minutes	CRP, troponin, CK andCK-MB levels	Remote ischemic preconditioning increased the release of cardiac enzymes
Karuppasamy 2011 [24]	On-pump CABG surgery	54	Remote ischemic preconditioning VS control	Arm ischemia and reperfusion, by inflation of a cuff at 200 mmHg placed at both arms, for 3×5 minutes	Troponin and CK-MB	Remote ischemic preconditioning did not significantly reduce troponin release
Li 2010 [27]	Valve surgery	81	Remote ischemic preconditioning VS remote ischemic perconditioning VS control	Leg ischemia and reperfusion, by inflation of a cuff at 600 mmHg placed at the leg, for 3×4 minutes	Clinical data and troponin levels	Remote perconditioning, but not preconditioning, reduced troponin levels
Luo 2011 [28]	VSD** correction.	60	Remote ischemic preconditioning VS direct postconditioning VS control	Leg ischemia and reperfusion, by inflation of a cuff at 200–300 mmHg placed at the leg, for 3×5 minutes	CK-MB and troponin levels, clinical data	Remote ischemic conditioning reduced biomarker release
Rahman 2010 [22]	On-pump CABG	162	Remote ischemic preconditioning VS control	Arm ischemia and reperfusion, by inflation of a cuff at 200 mmHg placed at the arm, for 3×5 minutes	Primary: troponin release	Remote ischemic conditioning did not reduce troponin levels
Rentoukas 2010 [32]	Primary PCI	96	Remote ischemic perconditioning VS remote ischemic perconditioning plus morphine VS control	Arm ischemia and reperfusion, by inflation of a cuff at 200 mmHg placed at the arm, for 3×5 minutes	Primary endpoint: achievement of full ST-segment resolution Secondary: peak troponin	Remote ischemic conditioning increased the achievement of ST-segment resolution
Thielmann 2010 [20]	On-pump CABG	53	Remote ischemic preconditioning VS control	Arm ischemia and reperfusion, by inflation of a cuff at 200 mmHg placed at the arm, for 3×5 minutes	Primary: Troponinlevels. Secondary:Mortality, major adversecardiovascular eventsand renal function	Remote ischemic conditioning reduced troponin release
Venugopal 2009 [17]	On-pump CABG, +/− AVR^#^	45	Remote ischemic preconditioning VS control	Arm ischemia and reperfusion, by inflation of a cuff at 200 mmHg placed at the arm, for 3×5 minutes	Troponin levels	Remote ischemic conditioning reduced troponin release
Venugopal 2010 [15]	On-pump CABG, +/− AVR (retrospective analysis of 2 prior studies)	78	Remote ischemic conditioning VS control	Arm ischemia and reperfusion, by inflation of a cuff at 200 mmHg placed at the arm, for 3×5 minutes	Kidney injury, defined as an increase of 25 umol/L	Remote ischemic conditioning reduced kidney injury
Wagner 2010 [19]	On-pump CABG, +/− valve surgery	101	Late remote ischemic preconditioning (18 hours before surgery) VS control VS tramadol	Arm ischemia and reperfusion, by inflation of a cuff at systolic pressure plus 40 mmHg placed at the arm, for 3×5 minutes	Troponin release	Remote ischemic conditioning reduced troponin release
Walsh 2009 [35]	Endovascular AAA repair	40	Remote ischemic preconditioning VS control	Leg ischemia and reperfusion, by inflation of a cuff placed at the thigh to a pressure that ensured absence of flow by echo-Doppler, 10 minutes of 1 leg,then 10 minutes of the other leg	Primary: kidney injury,measured byalbumin:creatinin ratioand retinol bindingprotein in urineSecondary: serumcreatinin and GFR	Remote ischemic conditioning did not significantly reduce kidney injury
Walsh 2010 [36]	Open AAA repair	40	Remote ischemic preconditioning VS control	Leg ischemia by clamping of the common iliac artery, 10 minutes left and 10 minutes right	Primary: kidney injury,measured byalbumin:creatinin ratioand retinol bindingprotein in urineSecondary: serumcreatinin and GFR	Remote ischemic conditioning did not reduce kidney injury
Walsh 2010 [37]	Carotid endarteriectomy	70	Remote ischemic conditioning VS control	Leg ischemia and reperfusion, by inflation of a cuff placed at the thigh to a pressure that ensured absence of flow by echo-Doppler, 10 minutes of 1 leg, then 10 minutes of the other leg	Primary: saccadic latency, troponin release Secondary: major adverse cardiovascular events	Remote ischemic conditioning did not significantly improve saccadic latency or troponin release
Zhou 2010 [26]	VSD repair	60	Combined late and early remote ischemic preconditioning VS control	Arm ischemia and reperfusion, by inflation of a cuff at 240 mmHg placed at the arm, for 3×5 minutes	Heart and lung function, inflammatory markers	Remote ischemic preconditioning had mixed effects on heart and lung function and inflammatory markers
Zimmerman 2011 [23]	On-pump cardiac surgery	60	Preconditioning VS no intervention	Leg ischemia and reperfusion, by inflation of surgical tourniquet to 200 mmHg placed at the thigh for 3×5 minutes	Kidney injury (increase of serum creatinine by >26.5 mmol/L)	Reduction in incidence of post-operative kidney injury by preconditioning

* abdominal aortic aneurysm; ^†^coronary artery bypass grafting; ^‡^percutaneous coronary intervention; ^§^left ventricular ejection fraction; ^||^major adverse cardiovascular event; ^#^aortic valve replacement; **ventricular septal defect.

#### Included studies

Most studies were done in patients undergoing cardiac surgery, including 10 studies in coronary artery bypass grafting surgery surgery, [16]–[24] and five in other cardiac surgery. [25]–[29] Four studies were done in patients undergoing percutaneous coronary intervention, of which two were elective [30], [31] and two were acute studies. [32], [33] Another four studies were done in patients undergoing vascular surgery. [34]–[37] Median trial size was 66 patients (range 8, 333 patients). Due to the small number of trials we only performed a sub-group analysis for the patients undergoing coronary artery bypass grafting surgery.

#### Conditioning stimulus

In most studies, remote ischemic conditioning was induced shortly before an expected period of ischemia (preconditioning). In two studies preconditioning was induced 18–24 hours before ischemia (late preconditioning), either by it self [19] or combined with early preconditioning. [26] One study [27] included a remote ischemic preconditioning group and a remote ischemic postconditioning group, as well as one control group. We treated these two groups as if they were from two separate studies, thereby including the control patients twice. In both primary PCI studies conditioning was induced during ischemia. [32], [33]

Remote ischemic conditioning was almost always induced by inflating a blood-pressure cuff or surgical tourniquet placed at one of the extremities. Only in two studies ischemia was induced by invasively clamping an artery. [34], [36] In 14 studies the arm was used to induce conditioning by three or four series of five minutes of ischemia and five minutes of reperfusion. [16]–[22], [24], [26], [30]–[33], [38] In nine studies the leg was used for conditioning by two or three series of five or ten minutes of ischemia. [23], [25], [27]–[29], [34]–[37]

### Quality of Studies

**Figure 2:**
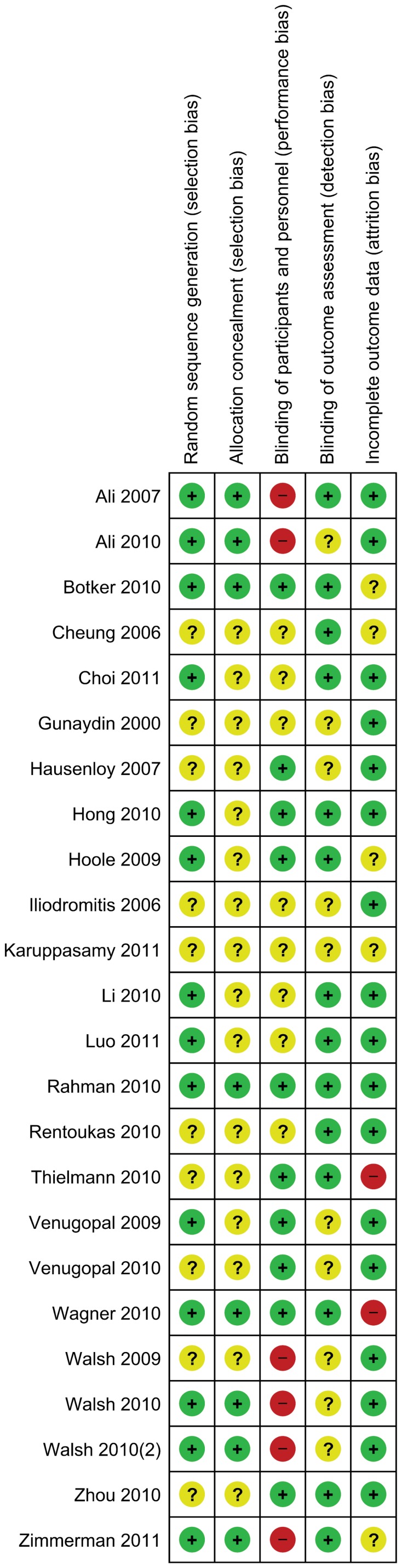
Risk of bias table: green = low risk of bias; yellow = unclear risk of bias; red = high risk of bias.

In one third of the studies the process of sequence generation and concealment was done properly, in the other studies the methods were not described in detail (see Figure 2). Adequate blinding was done in nine out of 23 studies. Six studies are at risk for performance bias, since the treating surgeon was not blinded for group allocation. This is in particular the case for the two studies in which the surgeon performed ischemic conditioning invasively. Nine studies were described as being blinded, but it was not specified how blinding of treating physicians was achieved. These studies were marked as having an unclear risk of bias. In one study, patients undergoing remote conditioning were sedated, while control patients were not. [26] Outcome assessment was blinded in two thirds of the studies, for the other studies it was not well described. Two studies excluded patients after randomization and might be at risk for attrition bias. [19], [20] One study recruited extra patients after the planned number of patients did not provide enough power, although the numbers in the article do not match the numbers in the flow-schedule. [38] In another study the patient numbers provided in text do not match those in the corresponding tables. [33] Finally, one study did not specify a primary outcome parameter and lacked a power calculation. [25]

### Effects of Remote Conditioning

#### Mortality and major adverse cardiovascular events

**Figure 3:**
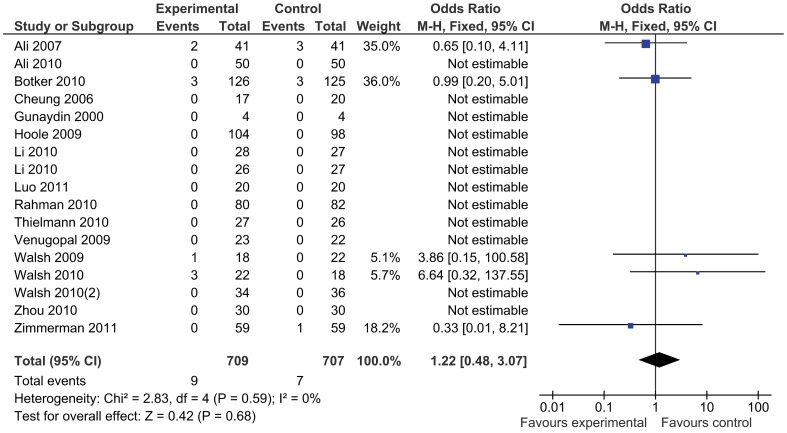
Mortality with remote ischemic conditioning and without remote ischemic conditioning.

**Figure 4:**
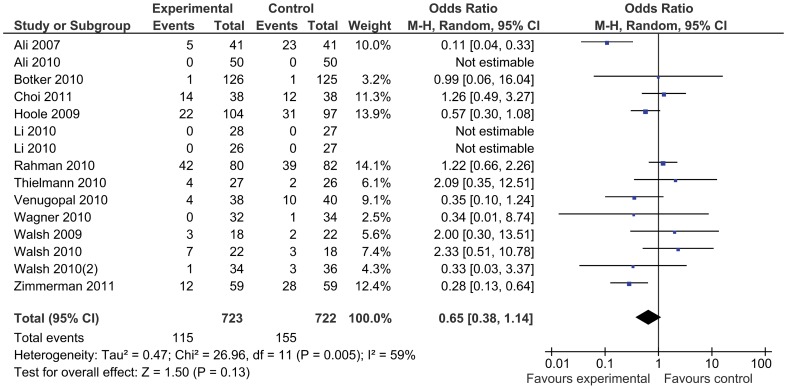
Major adverse cardiovascular events with remote ischemic conditioning and without remote ischemic conditioning.

**Figure 5:**
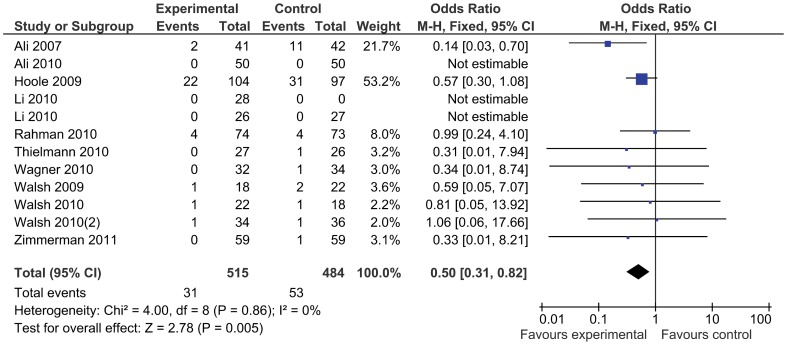
Myocardial infarction with remote ischemic conditioning and without remote ischemic conditioning.

In total, 954 patients were treated with remote ischemic conditioning and were compared with 924 control patients. Remote ischemic conditioning did not reduce the primary endpoint of mortality (Figure 3), which was reported in 16 studies and occurred in nine (1.3%) patients of 709 treated with remote ischemic conditioning, compared to seven (1.0%) out of 680 control patients (odds ratio 1.22 [95% confidence interval: 0.48, 3.07]). There was no evidence for statistical heterogeneity (I^2^ = 0%). The incidence of major adverse cardiovascular events was not reduced by remote conditioning (odds ratio 0.65 [0.38, 1.14], Figure 4), nor was the occurrence of atrial fibrillation (odds ratio 1.11 [0.63, 1.95]), or kidney injury (odds ratio 0.63 [0.28, 1.45]). Stroke was reported in five studies, but no events occurred. Remote ischemic conditioning reduced the risk of myocardial infarction (0.50 [0.31, 0.82]), with no statistical heterogeneity (I^2^ = 0%) (Figure 5). Including the studies with no events by adding one event in each study arm results in a recalculated odds ratio of 0.53 (0.34, 0.85). The l’Abbé plot shows two outliers: the studies Ali ’07 and Hoole ’09, a mid-size and large trial, both with a rather high incidence of infarctions. As a sensitivity analysis we recalculated the odds ratio without these studies, resulting in an odds ratio of 0.68 (0.28, 1.65). In the subgroup of patients undergoing coronary artery bypass grafting surgery there were four events in the intervention group compared to seven in the control group (odds ratio 0.63 [0.21, 1.89]).

#### Length of stay in hospital and in the intensive care unit

12 and 10 studies reported the length of stay in the hospital and in the intensive care unit, respectively, but both outcome parameters were not affected by remote ischemic conditioning (mean difference length-of-stay 0.04 days (95% confidence interval: −0.21, 0.29); mean difference intensive care unit stay −0.16 days (−0.38, 0.06), respectively).

#### Biomarkers

**Figure 6:**
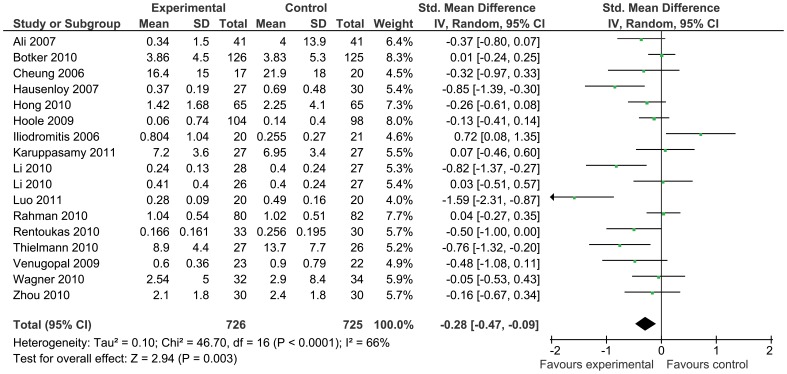
Peak troponin release with remote ischemic conditioning and without remote ischemic conditioning.

**Figure 7:**
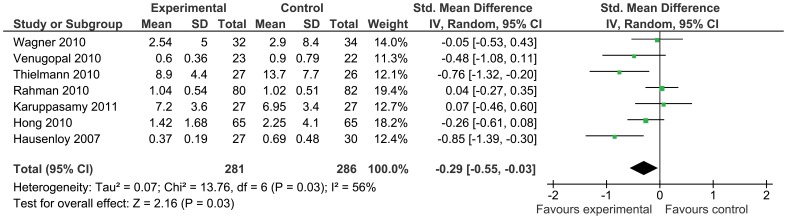
Peak troponin release with remote ischemic conditioning and without remote ischemic conditioning in the CABG-surgery sub-group.

Troponin was the most reported biomarker of myocardial injury and was significantly reduced by remote ischemic conditioning (standardized mean difference −0.28 [95% confidence interval: −0.47, −0.09], Figure 6), although there was significant statistical heterogeneity (I^2^ = 66%). Troponin release was also reduced in the coronary artery bypass grafting subgroup by remote ischemic conditioning as compared to control (standardized mean difference −0.29 [95% confidence interval −0.55, −0.03], Figure 7). Levels of creatine kinase were reported only in three studies (mean difference 19.54 U/L [−5.60, 44.69]), and the levels of creatine kinase muscle-brain in seven studies (mean difference −0.45 ug/L [−6.14, 5.24]), showing no difference between groups.

Regarding kidney function, creatinine clearance was reported in five studies and plasma creatinine levels in seven, which were both not reduced by remote ischemic conditioning (mean difference creatinine clearance 1.88 ml/min [−5.10, 8.87]; mean difference creatinine levels −6.50 mmol/L [−17.52, 4.53], respectively).

## Discussion

Despite including 23 studies the available evidence on the effect of remote ischemic conditioning on the clinical outcome of patients after ischemic events is limited, and strong conclusions can not be drawn. The present analysis does not support the hypothesis that remote ischemic conditioning reduces mortality associated with ischemic events, nor did remote conditioning reduce the combined endpoint of major adverse cardiovascular events. However, the secondary endpoint of periprocedural myocardial infarction was reduced by remote ischemic conditioning, as was the post-procedural peak release of troponin, although there are some limitations to these conclusions.

Most included clinical studies were rather small, and focused on the effect of remote conditioning on biomarker release and other surrogate parameters of organ injury. Short or long-term outcome was not the primary outcome variable in any study. Some studies did not report on the occurrence of adverse events, such as death or myocardial infarction. The overall mortality in studies that reported on this outcome was 1.2%, and the total number of events was 14, meaning that the incidence of the relevant event was quite low. It is debatable whether a reduction in mortality is possible in these patients. But to demonstrate an effect on mortality, if there is any, thousands of patients have to be included. For mortality the required group-size can not be calculated using our data, since we found no trend towards a reduction in mortality by remote conditioning. However, based on our data, with an incidence in the control group of 21%, 760 patients per group will be needed to detect a relative risk reduction of 27% in major adverse cardiovascular events, with an alpha of 0.05 and a beta of 0.80. Providing statistical significance would be reached, inevitably the question of the clinical impact of such findings would appear. The intervention of remote conditioning might prove to be more beneficial in high-risk patients; however, these high-risk patients were generally excluded from the clinical trials that were available for inclusion in the current meta-analysis.

Our analysis shows that remote conditioning reduces the incidence of myocardial infarction. When examining the endpoint of myocardial infarction we used the definition applied by the investigators of the respective studies. This means that different events could have been classified as myocardial infarctions, and we could have wrongly pooled these events in the meta-analysis. Additionally, the results of the meta-analysis are largely driven by the study of Ali et al. [34] In this study the control group had a rather high event rate of approximately 26% and might not represent the population as a whole. Also, the study intervention (invasive crossclamping of the iliac artery) was administered by the operating surgeon, which could have induced bias. In addition to a reduction in myocardial infarction, we also found that the release of biomarkers of myocardial injury was reduced after remote conditioning. In contrast, however, we found no reduction in mortality, or length of stay in hospital or in the intensive care unit. As mentioned in the meta-analysis of myocardial infarctions the studies by Hoole and Ali were assigned a high weight, of 53% and 22% respectively. The Hoole study was performed in low risk patients undergoing elective percutaneous coronary intervention, no mortalities occurred and could therefore not have been improved; length of stay was not reported. Ali et al did find a significant reduction in length of stay on the intensive care unit, as well as a non-significant reduction in length of stay in hospital. However, in the meta-analysis of these parameters their study was assigned a low weight and the effect was lost. This could explain why the reduction in myocardial infarctions does not translate into a reduction in length of stay, or mortality.

Troponin was commonly used as biomarker for myocardial injury. Quantities reported varied; some studies reported peak values, some area under the curve, while again other studies mentioned values at several time-points (which also differed between studies). We used peak values when reported, and otherwise approximated them as good as possible. However, this inevitably leads to variance since the reported time-points differed between studies and the extracted peak values varied greatly between studies, even in comparable settings. Therefore, it is uncertain whether our meta-analysis estimates the true effect of remote ischemic conditioning by missing the true peak values in some studies. In view of a resulting research agenda and as with other preconditioning studies, it would be advisable that the international research community establishes standards to measure organ injury in clinical studies.

An issue that troubles most studies is adequate blinding, which is probably more difficult to achieve than in trials investigating a pharmacological agent. Only in a minority of the trials all care providers were blinded, leaving the others at risk for bias.

In the present review we included studies with significant clinical heterogeneity. Studies differed in setting, patient population and the extent of ischemia the patients were at risk for. Patients undergoing cardiac surgery, vascular surgery, elective or acute percutaneous coronary intervention were all pooled for the meta-analysis on the chosen outcome parameters. We intended to perform sub-group analysis on special patient populations, but while analyzing the data it became obvious that only the group of studies in patients undergoing coronary artery bypass grafting was large enough to permit a sub-group analysis.

There is no uniform protocol to induce remote ischemic conditioning in the included studies. Different limbs are made ischemic and the duration and number of the ischemic periods differ. In a prior study Loukogeorgakis et al. [39] compared different regimes of remote postconditioning, comparing both arms and legs, and two and three cycles of five minutes ischemia and reperfusion. Their findings suggest that a threshold stimulus must be reached in order to achieve protection. Therefore, it might be that some studies used a form of conditioning which is not sufficient to achieve the maximal protective effect. Different kinds of anesthesia have been used in different clinical studies. From experimental and clinical studies it is known that pre- and postconditioning are influenced by different anesthetics. It is most likely that there is an effect of anesthetic regime on conditioning, and it would be interesting to perform an analysis based on different anesthetics used within our meta-analysis. However, most studies do not report the anesthetics used, and unless the anesthetic regime is specified in the protocol, it is impossible to identify this in retrospect. Therefore we chose not to perform such an analysis.

We used a random-effect model when statistical heterogeneity was high, and a fixed-effect model when it was low. Since random-effect models tend to provide a broader confidence interval than fixed-effect models, and can therefore be seen as more conservative, one may argue to use them for all analysis. This is not necessarily true, however, since random-effect models also put greater emphasis on small trials, and can be less conservative in those instances. [40] A comparison of the results of this approach and those of the respective random-effect model was performed, which did not alter the results (data not shown).

Recently Takagi and Umemoto published an update of their previous meta-analysis on remote ischemic preconditioning, [41] which confirmed their previously determined reduction in biomarker release. However, they found no reduction in mortality or peri-operative myocardial infarctions. Our findings are in line with their study, as we can show an effect on biomarker release, but no reduction in mortality. However, we did see a reduction in myocardial infarctions. There are a several differences between the study by Takagi and the present analysis, which might explain these different results. We included 23 studies compared to 9 studies with a total of 1878 patients compared to 482 included by Takagi. Next to a broader timeframe (until July 2011 versus April 2010) we also included a broader population of studies, encompassing four studies in patients undergoing a PCI. Concerning biomarker release, Takagi extracted different entities such as area-under-the-curve, peak values and single time-points, and subsequently pooled the data using the standardized mean differences, whereas we tried to extract peak values from all studies.

In summary, remote ischemic conditioning is an experimental technique and until today not part of accepted treatment protocols. Although studies on biomarker release show promising results, there is until today not enough evidence to recommend the routine use of remote ischemic conditioning to treat ischemic injury. Large trials are needed to investigate whether remote conditioning actually improves clinical outcomes. Research should also include high-risk patients, who might benefit most from protection by remote ischemic conditioning.

## References

[1] Murry CE, Jennings RB, Reimer KA (1986) Preconditioning with ischemia: a delay of lethal cell injury in ischemic myocardium. Circulation 74: 1124–36. 3769170 10.1161/01.cir.74.5.1124

[2] Hansen PR, Thibault H, Abdulla J (2010) Postconditioning during primary percutaneous coronary intervention: a review and meta-analysis. Int J Cardiol 144: 22–5 10.1016/j.ijcard.2009.03.118 19409628 10.1016/j.ijcard.2009.03.118

[3] Przyklenk K, Bauer B, Ovize M, Kloner RA, Whittaker P (1993) Regional ischemic ‘preconditioning’ protects remote virgin myocardium from subsequent sustained coronary occlusion. Circulation 87: 893–9. 7680290 10.1161/01.cir.87.3.893

[4] Kharbanda RK, Mortensen UM, White PA, Kristiansen SB, Schmidt MR, et al (2002) Transient limb ischemia induces remote ischemic preconditioning in vivo. Circulation 106: 2881–3. 12460865 10.1161/01.cir.0000043806.51912.9b

[5] Kerendi F, Kin H, Halkos ME, Jiang R, Zatta AJ, et al (2005) Remote postconditioning. Brief renal ischemia and reperfusion applied before coronary artery reperfusion reduces myocardial infarct size via endogenous activation of adenosine receptors. Basic Res Cardiol 100: 404–12 10.1007/s00395-005-0539-2 15965583 10.1007/s00395-005-0539-2

[6] Takagi H, Manabe H, Kawai N, Goto SN, Umemoto T (2008) Review and meta-analysis of randomized controlled clinical trials of remote ischemic preconditioning in cardiovascular surgery. Am J Cardiol 102: 1487–8 10.1016/j.amjcard.2008.07.036 19026301 10.1016/j.amjcard.2008.07.036

[7] Higgins JPT GS, ed. ( Cochrane Handbook for Systematic Reviews of Interventions Version 5.1.0 [updated March 2011]. : The Cochrane Collaboration, 2011. Available from. www.cochrane-handbook.org.

[8] Moher D, Liberati A, Tetzlaff J, Altman DG (2009) PRISMA Group (2009) Preferred reporting items for systematic reviews and meta-analyses: the PRISMA statement. BMJ 339: b2535. 19622551 10.1136/bmj.b2535 PMC2714657

[9] Review Manager (RevMan) [Computer program]. Version 5.1. Copenhagen: The Nordic Cochrane Centre, The Cochrane Collaboration.

[10] Hozo SP, Djulbegovic B, Hozo I (2005) Estimating the mean and variance from the median, range, and the size of a sample. BMC Med Res Methodol 5: 13 10.1186/1471-2288-5-13 15840177 10.1186/1471-2288-5-13 PMC1097734

[11] Hoole SP, Heck PM, White PA, Khan SN, O’Sullivan M, et al (2009) Remote ischemic preconditioning stimulus does not reduce microvascular resistance or improve myocardial blood flow in patients undergoing elective percutaneous coronary intervention. Angiology 60: 403–11 10.1177/0003319708328921 19106155 10.1177/0003319708328921

[12] Hoole SP, Khan SN, White PA, Heck PM, Kharbanda RK, et al (2009) Remote ischaemic pre-conditioning does not attenuate ischaemic left ventricular dysfunction in humans. Eur J Heart Fail 11: 497–505 10.1093/eurjhf/hfp040 19386814 10.1093/eurjhf/hfp040

[13] Hu S, Dong HL, Li YZ, Luo ZJ, Sun L, et al (2010) Effects of remote ischemic preconditioning on biochemical markers and neurologic outcomes in patients undergoing elective cervical decompression surgery: a prospective randomized controlled trial. J Neurosurg Anesthesiol 22: 46–52 10.1097/ANA.0b013e3181c572bd 19996767 10.1097/ANA.0b013e3181c572bd

[14] Munk K, Andersen NH, Schmidt MR, Nielsen SS, Terkelsen CJ, et al (2010) Remote Ischemic Conditioning in Patients With Myocardial Infarction Treated With Primary Angioplasty: Impact on Left Ventricular Function Assessed by Comprehensive Echocardiography and Gated Single-Photon Emission CT. Circulation. Cardiovascular imaging 3: 656–62 10.1161/CIRCIMAGING.110.957340 20826592 10.1161/CIRCIMAGING.110.957340

[15] Venugopal V, Laing CM, Ludman A, Yellon DM, Hausenloy D (2010) Effect of remote ischemic preconditioning on acute kidney injury in nondiabetic patients undergoing coronary artery bypass graft surgery: a secondary analysis of 2 small randomized trials. Am J Kidney Dis 56: 1043–9 10.1053/j.ajkd.2010.07.014 20974511 10.1053/j.ajkd.2010.07.014 PMC2991586

[16] Hausenloy DJ, Mwamure PK, Venugopal V, Harris J, Barnard M, et al (2007) Effect of remote ischaemic preconditioning on myocardial injury in patients undergoing coronary artery bypass graft surgery: a randomised controlled trial. Lancet 370: 575–9 10.1016/S0140-6736(07)61296-3 17707752 10.1016/S0140-6736(07)61296-3

[17] Venugopal V, Hausenloy DJ, Ludman A, Di Salvo C, Kolvekar S, et al (2009) Remote ischaemic preconditioning reduces myocardial injury in patients undergoing cardiac surgery with cold-blood cardioplegia: a randomised controlled trial. Heart 95: 1567–71 10.1136/hrt.2008.155770 19508973 10.1136/hrt.2008.155770

[18] Günaydin B, Cakici I, Soncul H, Kalaycioglu S, Cevik C, et al (2000) Does remote organ ischaemia trigger cardiac preconditioning during coronary artery surgery? Pharmacol Res 41: 493–6 10.1006/phrs.1999.0611 10704275 10.1006/phrs.1999.0611

[19] Wagner R, Piler P, Bedanova H, Adamek P, Grodecka L, et al (2010) Myocardial injury is decreased by late remote ischaemic preconditioning and aggravated by tramadol in patients undergoing cardiac surgery: a randomised controlled trial. Interact Cardiovasc Thorac Surg 11: 758–62 10.1510/icvts.2010.243600 20847065 10.1510/icvts.2010.243600

[20] Thielmann M, Kottenberg E, Boengler K, Raffelsieper C, Neuhaeuser M, et al (2010) Remote ischemic preconditioning reduces myocardial injury after coronary artery bypass surgery with crystalloid cardioplegic arrest. Basic Res Cardiol 105: 657–64 10.1007/s00395-010-0104-5 20495811 10.1007/s00395-010-0104-5

[21] Ali N, Rizwi F, Iqbal A, Rashid A (2010) Induced remote ischemic pre-conditioning on ischemia-reperfusion injury in patients undergoing coronary artery bypass. J Coll Physicians Surg Pak 20: 427–31 07.2010/JCPSP.427431 20642939

[22] Rahman IA, Mascaro JG, Steeds RP, Frenneaux MP, Nightingale P, et al (2010) Remote ischemic preconditioning in human coronary artery bypass surgery: from promise to disappointment? Circulation 122: S53–9 10.1161/CIRCULATIONAHA.109.926667 20837926 10.1161/CIRCULATIONAHA.109.926667

[23] Zimmerman RF, Ezeanuna PU, Kane JC, Cleland CD, Kempananjappa TJ, et al (2011) Ischemic preconditioning at a remote site prevents acute kidney injury in patients following cardiac surgery. Kidney Int 80: 861–7 10.1038/ki.2011.156 21677633 10.1038/ki.2011.156

[24] Karuppasamy P, Chaubey S, Dew T, Musto R, Sherwood R, et al (2011) Remote intermittent ischemia before coronary artery bypass graft surgery: a strategy to reduce injury and inflammation? Basic Res Cardiol 106: 511–9 10.1007/s00395-011-0185-9 21544683 10.1007/s00395-011-0185-9

[25] Cheung MMH, Kharbanda RK, Konstantinov IE, Shimizu M, Frndova H, et al (2006) Randomized controlled trial of the effects of remote ischemic preconditioning on children undergoing cardiac surgery: first clinical application in humans. J Am Coll Cardiol 47: 2277–82 10.1016/j.jacc.2006.01.066 16750696 10.1016/j.jacc.2006.01.066

[26] Zhou W, Zeng D, Chen R, Liu J, Yang G, et al (2010) Limb ischemic preconditioning reduces heart and lung injury after an open heart operation in infants. Pediatr Cardiol 31: 22–9 10.1007/s00246-009-9536-9 19787388 10.1007/s00246-009-9536-9

[27] Li L, Luo W, Huang L, Zhang W, Gao Y, et al (2010) Remote perconditioning reduces myocardial injury in adult valve replacement: a randomized controlled trial. J Surg Res 164: e21–6 10.1016/j.jss.2010.06.016 20850778 10.1016/j.jss.2010.06.016

[28] Luo W, Zhu M, Huang R, Zhang Y (2011) A comparison of cardiac post-conditioning and remote pre-conditioning in paediatric cardiac surgery. Cardiol Young: 1–5 10.1017/S1047951110001915 10.1017/S1047951110001915 21262079

[29] Choi YS, Shim JK, Kim JC, Kang KS, Seo YH, et al (2011) Effect of remote ischemic preconditioning on renal dysfunction after complex valvular heart surgery: a randomized controlled trial. J Thorac Cardiovasc Surg 142: 148–54 10.1016/j.jtcvs.2010.11.018 21272897 10.1016/j.jtcvs.2010.11.018

[30] Iliodromitis EK, Kyrzopoulos S, Paraskevaidis IA, Kolocassides KG, Adamopoulos S, et al (2006) Increased C reactive protein and cardiac enzyme levels after coronary stent implantation. Is there protection by remote ischaemic preconditioning? Heart 92: 1821–6 10.1136/hrt.2006.089060 16855045 10.1136/hrt.2006.089060 PMC1861265

[31] Hoole SP, Heck PM, Sharples L, Khan SN, Duehmke R, et al (2009) Cardiac Remote Ischemic Preconditioning in Coronary Stenting (CRISP Stent) Study: a prospective, randomized control trial. Circulation 119: 820–7 10.1161/CIRCULATIONAHA.108.809723 19188504 10.1161/CIRCULATIONAHA.108.809723

[32] Rentoukas I, Giannopoulos G, Kaoukis A, Kossyvakis C, Raisakis K, et al (2010) Cardioprotective role of remote ischemic periconditioning in primary percutaneous coronary intervention: enhancement by opioid action. JACC. Cardiovascular interventions 3: 49–55 10.1016/j.jcin.2009.10.015 20129568 10.1016/j.jcin.2009.10.015

[33] Bøtker HE, Kharbanda R, Schmidt MR, Bøttcher M, Kaltoft AK, et al (2010) Remote ischaemic conditioning before hospital admission, as a complement to angioplasty, and effect on myocardial salvage in patients with acute myocardial infarction: a randomised trial. Lancet 375: 727–34 10.1016/S0140-6736(09)62001-8 20189026 10.1016/S0140-6736(09)62001-8

[34] Ali ZA, Callaghan CJ, Lim E, Ali AA, Nouraei SAR, et al (2007) Remote ischemic preconditioning reduces myocardial and renal injury after elective abdominal aortic aneurysm repair: a randomized controlled trial. Circulation 116: I98–105 10.1161/circulationaha.106.679167 17846333 10.1161/circulationaha.106.679167

[35] Walsh SR, Boyle JR, Tang TY, Sadat U, Cooper DG, et al (2009) Remote ischemic preconditioning for renal and cardiac protection during endovascular aneurysm repair: a randomized controlled trial. J Endovasc Ther 16: 680–9 10.1583/09-2817.1 19995115 10.1583/09-2817.1

[36] Walsh SR, Sadat U, Boyle JR, Tang TY, Lapsley M, et al (2010) Remote ischemic preconditioning for renal protection during elective open infrarenal abdominal aortic aneurysm repair: randomized controlled trial. Vasc Endovascular Surg 44: 334–40 10.1177/1538574410370788 20484066 10.1177/1538574410370788

[37] Walsh SR, Nouraei SA, Tang TY, Sadat U, Carpenter RH, et al (2010) Remote ischemic preconditioning for cerebral and cardiac protection during carotid endarterectomy: results from a pilot randomized clinical trial. Vasc Endovascular Surg 44: 434–9 10.1177/1538574410369709 20484064 10.1177/1538574410369709

[38] Hong DM, Mint JJ, Kim JH, Sohn IS, Lim TW, et al (2010) The effect of remote ischaemic preconditioning on myocardial injury in patients undergoing off-pump coronary artery bypass graft surgery. Anaesth Intensive Care 38: 924–9. 20865880 10.1177/0310057X1003800518

[39] Loukogeorgakis SP, Williams R, Panagiotidou AT, Kolvekar SK, Donald A, et al (2007) Transient limb ischemia induces remote preconditioning and remote postconditioning in humans by a K(ATP)-channel dependent mechanism. Circulation 116: 1386–95 10.1161/CIRCULATIONAHA.106.653782 17724264 10.1161/CIRCULATIONAHA.106.653782

[40] Kranke P (2010) Evidence-based practice: how to perform and use systematic reviews for clinical decision-making. Eur J Anaesthesiol 27: 763–72 10.1097/EJA.0b013e32833a560a 20523217 10.1097/EJA.0b013e32833a560a

[41] Takagi H, Umemoto T (2011) Remote ischemic preconditioning for cardiovascular surgery: an updated meta-analysis of randomized trials. Vasc Endovascular Surg 45: 511–3 10.1177/1538574410379654 21646235 10.1177/1538574410379654

